# Significance of cervical secretion culture in predicting maternal and fetal outcome in pregnant women with premature rupture of membranes: a retrospective cohort study

**DOI:** 10.3389/fphar.2024.1328107

**Published:** 2024-02-22

**Authors:** Zhenna Wang, Xiaoyan Xiu, Liying Zhong, Yi Wang, Zhuanji Fang, Shunhe Lin, Huihui Huang

**Affiliations:** Department of Obstetrics and Gynecology, Fujian Maternity and Child Health Hospital, College of Clinical Medicine for Obstetrics & Gynecology and Pediatrics, Fujian Medical University, Fuzhou, China

**Keywords:** premature rupture of membranes, chorioamnionitis, cervical secretion cultures, retrospective cohort study, pregnant women

## Abstract

**Background:** To investigate the clinical value of cervical secretion culture in pregnant women with premature rupture of membranes (PROM) in predicting maternal and fetal outcomes.

**Methods:** We retrospectively reviewed clinical records of pregnant women who underwent obstetric examination and delivered in Fujian Maternal and Child Healthcare from December 2013 to December 2016. Pregnant women with a clear diagnosis of PROM, who underwent cervical secretion culture immediately after hospital admission were selected for the study. The primary outcome was the occurrence of chorioamnionitis. The secondary outcome was neonatal admission to the neonatal intensive care unit (NICU). Correlation between maternal and fetal outcomes and the results of the cervical secretion culture was analyzed by one-way analysis and multifactorial analysis, respectively. The predictive efficacy of cervical secretion culture was evaluated using receiver operating characteristic curve (ROC), area under the curve (AUC) and the integrated discrimination improvement (IDI).

**Results:** A total of 7,727 pregnant women with PROM were included in the study. Of them, 1812 had positive cervical secretion cultures (635 positive for *mycoplasma* infection, 475 for bacterial, 637 for fungal, and 65 for chlamydial infections). Pregnant women with positive *mycoplasma* and bacterial cultures had higher rates of developing chorioamnionitis compared to women with negative cervical secretion cultures (9%, 12% vs. 1%, respectively). Similarly, positive *mycoplasma* and bacterial cultures were associated with higher rate of the preterm (before 34 weeks) labor (3%, 3% vs. 1% in women with negative cultures, respectively), and neonatal admission to the NICU (9%, 11% vs. 7%, respectively). After adjusting for various confounding factors, our analysis demonstrated that a positive cervical secretion culture for *mycoplasma* or bacterial pathogens remained an independent risk factor for chorioamnionitis. Cervical secretion culture outcome was less effective in predicting chorioamnionitis (AUC 0.569) compared to white blood count (WBC) (AUC 0.626) and C-reactive protein (CRP) levels (AUC 0.605). The IDI of the combined predictive model incorporating WBC, CRP, maternal fever and cervical secretion culture results was 0.0029^.^

**Conclusion:** Positive cervical secretion cultures, especially for *mycoplasma* and bacteria, are associated with higher incidence of adverse maternal and fetal outcomes. However, the predictive value of this test is poor, and cannot be efficiently used for predicting chorioamnionitis.

## Introduction

Premature rupture of membranes (PROM) is a common obstetric complication that may lead to adverse pregnancy outcomes such as placental abruption, maternal and fetal infections, and neonatal asphyxia (Prelabor Rupture of Membranes: ACOG Practice Bulletin, Number 217, 2020). Among these complications, chorioamnionitis is particularly notable for its long-term adverse effects on the neonate, such as neonatal sepsis and abnormal neurological development (Jain et al., 2022; Tsamantioti and Razaz, 2022; Yamaguchi-Goto et al., 2023). Yet, early clinical features of chorioamnionitis are not clear, and the diagnosis is often made only after the onset of maternal symptoms (fever, uterine fundal tenderness, tachycardia) and fetal distress, which leads to more serious adverse effects. Therefore, it is particularly important to monitor pregnant women with PROM and intervene in a timely manner. However, there is still a lack of good predictive methods for chorioamnionitis that are non-invasive, easy to perform, specific and effective in clinical practice.

Numerous studies have shown that PROM is closely related to reproductive tract infections that are detected by microbiological cultures of cervical secretions (Xu et al., 2022; Liu et al., 2023; Taylor et al., 2023). Cervical secretion culture is safe and easy to perform, and may detect subclinical chorioamnionitis at an early stage. The results of the cervical secretion culture together with the drug sensitivity test may be used to guide a subsequent antibiotic treatment. Previous studies have shown that the occurrence of chorioamnionitis may be predicted by measuring maternal inflammatory indicators in combination with the results of the cervical secretion cultures (Huang et al., 2022; Berg et al., 2023; Zhang et al., 2023). This study aims to analyze the correlation between the results of the cervical secretion culture and pregnancy and neonatal outcomes in women with PROM. Our results may provide evidence for the efficacy of cervical secretion culture in predicting chorioamnionitis and maternal and fetal outcomes, and to clarify its application value in PROM complicated by chorioamnionitis.

## Materials and methods

### Study design

**FIGURE 1:**
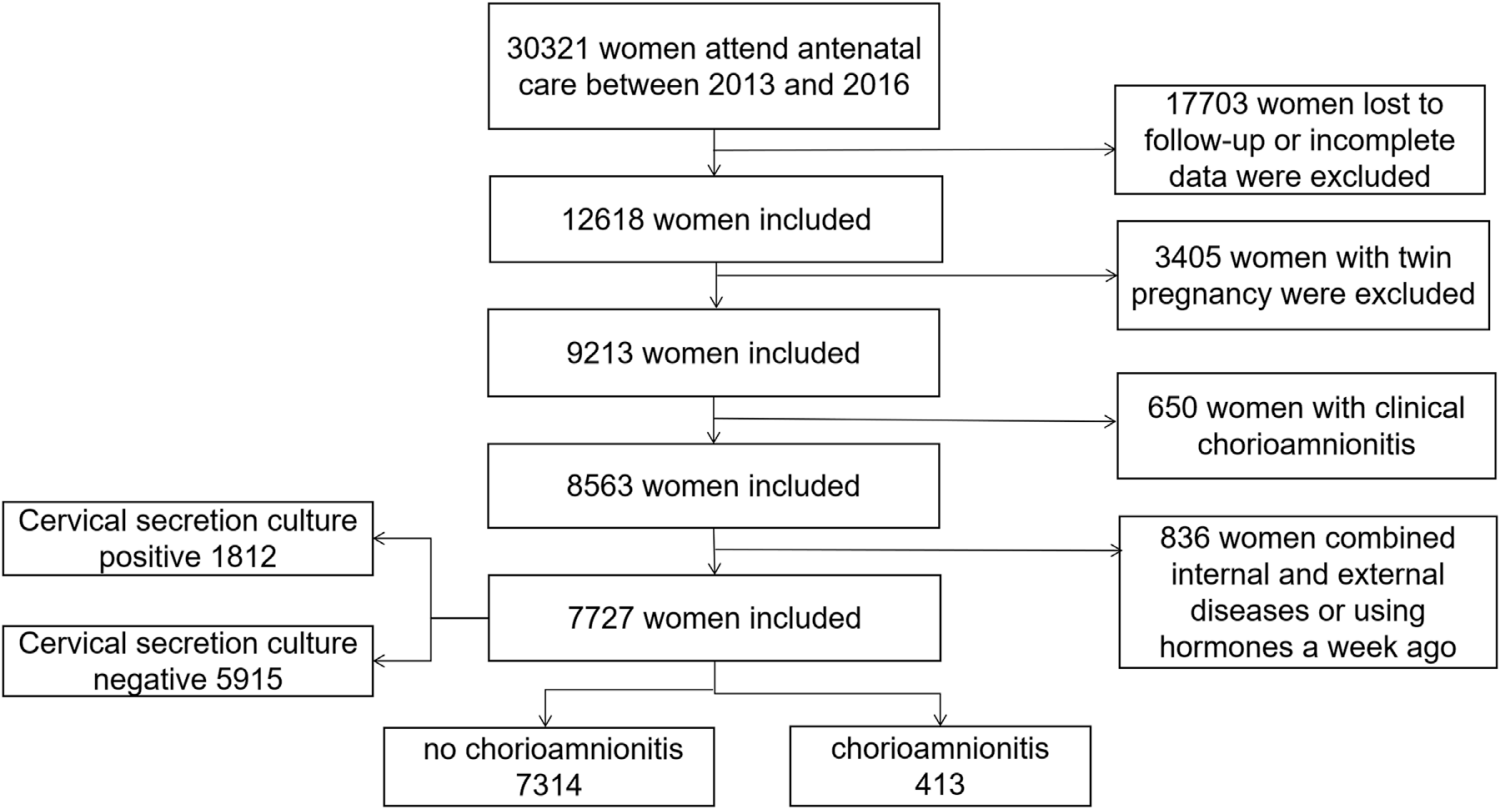
Flowchart of participants selection for the cohort.

We conducted a large-scale population-based retrospective cohort study of pregnant women who had regular medical check-ups and delivered at Fujian Maternal and Child Health Hospitals from December 2013 to December 2016, and 7,727 pregnant women with preterm premature rupture of membranes were ultimately enrolled in the study (Figure 1). All participants underwent routine blood counts and C-reactive protein (CRP) levels measurements in the peripheral blood. Cervical secretion cultures were done immediately after admission to the hospital, and antibiotics were administered to prevent infections when preterm rupture of membranes was present for more than 12 h. Placentas were sent for pathology for confirmation of the diagnosis of chorioamnionitis.

This study was approved by the Ethics Committee of Fujian Maternal and Child Health Hospital (#2022KYLLR0102). Due to the retrospective nature of the study, a written informed consent from the patients was not required.

### Eligibility criteria

Inclusion criteria were as follows: 1) Premature rupture of membranes, diagnosed according to the Clinical Practice Guidelines for Premature Rupture of Fetal Membranes (2020)" published by the American College of Obstetricians and Gynecologists (ACOG) in 2020 [1]; 2) Chorioamnionitis: infiltration of more than 5 neutrophils per high power field of view in pathological tissue of placenta, membranes, and umbilical cord.

Exclusion criteria: 1) Immediate delivery on admission, before the cervical secretion cultures was taken; 2) Typical clinical symptoms of chorioamnionitis (including elevated temperature, increased fetal heart rate, increased white blood cell count, increased pulse rate, etc.) on admission; 3) Use of antibiotics or glucocorticosteroids, etc. in the week prior to admission; 4) Failure to obtain the pathological results of placenta; 5) Comorbidities, such as upper respiratory tract infections, pneumonia, acute appendicitis, acute gastroenteritis and acute cholecystitis, etc.; and 6) Lost to follow up.

### Methods

Clinical information on pregnant women was collected through the hospital’s case system and included:

1) General information: age at delivery, education, maternal history, history of previous preterm labor, height, maternal fever (axillary temperature >37.8°C), pre-pregnancy weight and body mass index;
2) Laboratory indicators: routine blood indicators, including white blood count (WBC), neutrophil count (NE), levels of CRP;
3) Cervical secretion cultures. Cervical secretion cultures were done as follows: pregnant women were placed in the cystotomy position, vulva was disinfected, cervix exposed by speculum. The outer cervical orifice was swabbed by the sterile cotton swab. Cotton swab was inserted 1–2 cm into the cervical canal, rotated 5 times for a total of 20 s, and the secretions were cultured within 2 h. Swabs were inoculated into petri dishes and placed in 37°C, 5% CO2 for 24–48 h before checking to select target colonies for further microbial identification. The identification was carried out using the French bioMérieux VITEK-2 bacterial fully automatic identification analyser, and the results were interpreted according to the CLSI 2014 edition. The quality control strains were *Escherichia coli* ATCC 25922, *Pseudomonas aeruginosa* ATCC 27853, *Staphylococcus aureus* ATCC 29213, and *Enterococcus faecalis* ATCC 29212, all of which were purchased from the Clinical Inspection Centre of the Ministry of Health. The culture mediums were Columbia Blood Plate and Shap Paul Plate, and the *Candida* Comacia chromogenic medium was the product of Berrett Biotechnology (Zhengzhou) Limited Liability Company.
4) Maternal pregnancy outcome: preterm delivery, preterm delivery <34 weeks, mode of delivery, amniotic fluid properties, duration of the first stage of labor, post-partum hemorrhage, gestational diabetes mellitus, precipitous delivery, gestational hypertension, oligohydramnios, polyhydramnios, placental adhesions, placenta implantation, cervical insufficiency, placenta previa, placental abruptio placenta, puerperal infections, chorionic villus amnio amnionitis;
5) Neonatal outcome: Apgar score, neonatal hyperbilirubinemia, less than gestational age neonates, neonatal asphyxia, and NICU admission.

### Statistical analysis

R software (4.1.1) was used for the analysis, and the normality test was performed on the measurement data, with the mean ± standard deviation, expressed as X ± SD, for data that met the normal distribution. Independent samples t-test was used to compare the differences between the two groups. Non-normally distributed data were taken as non-parametric rank sum test. Count data were described by frequency (percentage), and differences between groups were compared using the chi-square test or Fisher’s exact test. Logistic regression models were used to correct for confounding factors such as age, cervical secretion cultures, and gestational diabetes mellitus, and ratios and 95% confidence intervals (CIs) were calculated. The predictive efficacy of cervical secretion cultureswas evaluated using receiver operating characteristic curve (ROC), area under the curve (AUC) and the integrated discrimination improvement (IDI). *p* < 0.05 was statistically different.

## Results

### Subgroup comparisons based on the results of cervical secretion cultures

A total of 7,727 pregnant women with PROM were included in the study (Figure 1), of whom 1812 had positive cervical secretion cultures. Baseline characteristics of study participants are summarized in Table 1. The average age of pregnant women at delivery was 28 years. There was no statistical difference between the groups in terms of maternal education, number of pregnancies and births, previous history of preterm labor, and pre-pregnancy BMI (*p* > 0.05). WBC, NE and CRP counts were significantly higher in the mycoplasma-, bacteria-, fungal- and chlamydia-positive groups, compared to the infection-negative group (*p* < 0.05). In terms of maternal pregnancy outcome, there was no significant difference in the incidence of complicated gestational diabetes mellitus, oligohydramnios, placenta previa, gestational diabetes mellitus, and post-partum hemorrhage (Table 1, *p* > 0.05). However, there was a statistically significant difference between the groups in the volume of post-partum hemorrhage (mL), the number of preterm deliveries at < 34 weeks, the mode of delivery, the amniotic fluid degree II-III turbidity, gestational hypertension, complicated puerperal infections, and chorionic amnio-amnionitis (Table 1, *p* < 0.05). Stratifying the gestational weeks of delivery revealed statistically significant intergroup association between cervical culture results and the rate of difficult miscarriages and deliveries at full-term.

**TABLE 1:** Clinical Baseline and Maternal Pregnancy Outcomes based on the Cervical Secretion Culture results.

Culture results/Characteristics	Negative (n = 5,915)	mycoplasma (n = 635)	bacterial (n = 475)	Fungal (n = 637)	chlamydia (n = 65)	*p*-Value
Age at delivery (y)	28 (26.32)	28 (26.32)	28 (26.31)	28 (26.32)	28 (26.32)	0.002
Education level [n (%)]						0.987
Primary school or below [n (%)]	41 (1)	5 (1)	4 (1)	4 (1)	0 (0)	
Junior and Senior high school [n (%)]	1,235 (25)	148 (25)	101 (25)	126 (23)	12 (23)	
College or higher [n (%)]	3,643 (74)	430 (74)	300 (74)	418 (76)	41 (77)	
Gravity						0.481
G < 3	4,340 (73)	475 (75)	370 (78)	478 (75)	47 (72)	
3 ≤ G < 5	1,357 (23)	136 (21)	92 (19)	133 (21)	17 (26)	
G ≥ 5	218 (4)	24 (4)	13 (3)	26 (4)	1 (2)	
Parity [n (%)]						0.159
0	3,525 (60)	411 (65)	296 (62)	378 (59)	41 (63)	
1	2,240 (38)	211 (33)	173 (36)	241 (38)	23 (35)	
2	144 (2)	13 (2)	5 (1)	17 (3)	1 (2)	
3	6 (0)	0 (0)	0 (0)	1 (0)	0 (0)	
4	0 (0)	0 (0)	1 (0)	0 (0)	0 (0)	
Previous history of premature birth [n (%)]						0.18
0	5,774 (98)	622 (98)	464 (98)	612 (96)	63 (97)	
1	141 (2)	13 (2)	11 (2)	25 (4)	2 (3)	
Pre-pregnancy BMI (kg/m^2^)	20.31 (18.81, 22.1)	20.31 (18.82, 22.2)	20.62 (19.1, 22.6)	20.31 (18.59, 22.26)	19.9 (18.52, 22.68)	0.092
Apgar score	10 (10, 10)	10 (10, 10)	10 (10, 10)	10 (10, 10)	10 (10, 10)	0.491
premature birth (<37 w) [n (%)]						0.66
0	5,342 (90)	563 (89)	428 (90)	568 (89)	59 (91)	
1	573 (10)	72 (11)	47 (10)	69 (11)	6 (9)	
Premature birth (<34 w) [n (%)]						<0.001
0	5,838 (99)	614 (97)	461 (97)	629 (99)	65 (100)	
1	77 (1)	21 (3)	14 (3)	8 (1)	0 (0)	
Delivery method [n (%)]						<0.001
CS	1,300 (22)	183 (29)	148 (31)	152 (24)	18 (28)	
VB	4,615 (78)	452 (71)	327 (69)	485 (76)	47 (72)	
Amniotic fluid II, III degree [n (%)]						<0.001
0	5,211 (88)	541 (85)	391 (82)	570 (89)	52 (80)	
1	704 (12)	94 (15)	84 (18)	67 (11)	13 (20)	
Amount of postpartum hemorrhage (mL)	175 (145, 290)	185 (145, 322.5)	195 (150, 324.25)	185 (150, 311.5)	190 (150, 320)	<0.001
Postpartum hemorrhage						0.622
0	5,807 (98)	618 (97)	466 (98)	624 (98)	64 (98)	
1	108 (2)	17 (3)	9 (2)	13 (2)	1 (2)	
Precipitate labour						0.004
0	5,300 (90)	580 (91)	449 (95)	583 (92)	61 (94)	
1	615 (10)	55 (9)	26 (5)	54 (8)	4 (6)	
GDM [n (%)]						0.176
0	6,367 (82)	4,869 (82)	534 (84)	376 (79)	536 (84)	
1	1,360 (18)	1,046 (18)	101 (16)	99 (21)	101 (16)	
HDP [n (%)]						0.011
0	5,735 (97)	615 (97)	448 (94)	618 (97)	60 (92)	
1	180 (3)	20 (3)	27 (6)	19 (3)	5 (8)	
Oligohydramnios [n (%)]						
0	5,891 (100)	633 (100)	467 (98)	636 (100)	65 (100)	5,891 (100)
1	24 (0)	2 (0)	8 (2)	1 (0)	0 (0)	24 (0)
Polyhydramnios [n (%)]						0.048
0	5,900 (100)	632 (100)	471 (99)	634 (100)	64 (98)	
1	15 (0)	3 (0)	4 (1)	3 (0)	1 (2)	
Placenta previa [n (%)]						0.067
0	5,896 (100)	630 (99)	470 (99)	634 (100)	65 (100)	
1	19 (0)	5 (1)	5 (1)	3 (0)	0 (0)	
Placental abruption [n (%)]						0.083
0	5,834 (99)	620 (98)	472 (99)	624 (98)	65 (100)	
1	81 (1)	15 (2)	3 (1)	13 (2)	0 (0)	
Puerperal infection [n (%)]						0.003
0	5,914 (100)	632 (100)	473 (100)	637 (100)	65 (100)	
1	1 (0)	3 (0)	2 (0)	0 (0)	0 (0)	
Chorioamnionitis [n (%)]						<0.001
0	5,642 (95)	580 (91)	420 (88)	610 (96)	62 (95)	
1	273 (5)	55 (9)	55 (12)	27 (4)	3 (5)	
Neonatal jaundice [n (%)]						0.016
0	3,945 (67)	454 (71)	341 (72)	443 (70)	41 (63)	
1	1970 (33)	181 (29)	134 (28)	194 (30)	24 (37)	
SGA [n (%)]						0.629
0	5,733 (97)	619 (97)	466 (98)	617 (97)	64 (98)	
1	182 (3)	16 (3)	9 (2)	20 (3)	1 (2)	
Mild asphyxia [n (%)]						0.674
0	5,895 (100)	633 (100)	472 (99)	634 (100)	65 (100)	
1	20 (0)	2 (0)	3 (1)	3 (0)	0 (0)	
Serve asphyxia [n (%)]						0.185
0	5,911 (100)	635 (100)	474 (100)	635 (100)	65 (100)	
1	4 (0)	0 (0)	1 (0)	2 (0)	0 (0)	
NICU [n (%)]						0.003
0	5,512 (93)	575 (91)	424 (89)	585 (92)	60 (92)	
1	403 (7)	60 (9)	51 (11)	52 (8)	5 (8)	
WBC	9.33 (7.83, 11.25)	9.73 (8.1, 11.5)	9.81 (8.17, 11.89)	9.57 (8.06, 11.45)	10 (8.21, 12.27)	<0.001
NE	6.82 (5.54, 8.51)	7.26 (5.93, 8.87)	7.2 (5.78, 9.02)	7.01 (5.62, 8.76)	6.88 (5.73, 8.83)	<0.001
CRP	2.15 (0.8, 4.96)	2.6 (1.01, 8.05)	2.9 (1.22, 9.11)	2.46 (0.78, 6.12)	2.5 (1.3, 5.52)	<0.001
Disease Type [n (%)]						0.001
extremely preterm (<28w)	8 (0)	2 (0)	0 (0)	2 (0)	0 (0)	
very preterm (<32w)	29 (0)	9 (1)	12 (3)	4 (1)	0 (0)	
Moderate preterm (32-33w)	40 (1)	10 (2)	2 (0)	2 (0)	0 (0)	
late preterm (34-36w)	496 (8)	51 (8)	33 (7)	61 (10)	6 (9)	
term labor (>37w)	5,342 (90)	563 (89)	428 (90)	568 (89)	59 (91)	
Cervical secretion culture [n (%)]						<0.001
0	5,915 (100)	0 (0)	0 (0)	0 (0)	0 (0)	
1	0 (0)	635 (100)	475 (100)	637 (100)	65 (100)	
Membrane rupture to delivery time(h)	32.76 (19.48, 45.65)	34.11 (21.24, 46.04)	34.4 (22.32, 46.66)	32.23 (19.16, 44.16)	29.38 (19.4, 45.33)	0.043
Fever (maternal temperature), n (%)						0.007
0	5,875 (99)	625 (98)	467 (98)	628 (99)	64 (98)	
1	40 (1)	10 (2)	8 (2)	9 (1)	1 (2)	

BMI, body mass index; WBC, white blood cell count; NE, neutrophil count; CRP, C-reactive protein; G, gravity; GDM, gestational diabetes mellitus; HDP, hypertension disorders of pregnancy; CS, cesarean section; VB, vaginal delivery; SGA, small for gestational age.

*Mycoplasma* and bacterial infections were associated with higher rate of preterm deliveries at < 34 w (3%, 3% VS. 1% in women with negative cultures, respectively), and concomitant chorionic amnioamnionitis (9%, 12%, respectively VS. 5% in the negative group). In terms of neonatal outcomes, there were no significant differences between the groups (*p* > 0.05) in the incidences of less than gestational age neonates, neonatal asphyxia, and Apgar scores, whereas the difference between the rates of neonatal hyperbilirubinemia in positive- and negative groups were statistically significant (*p* < 0.05). Cervical secretion cultures, positive for *mycoplasma*, bacteria, fungi, and *chlamydia*, were associated with higher rate of NICU admissions compared to the negative group (9%, 11%, 8%, 8% VS. 7%, respectively).

### Comparison of maternal and neonatal outcomes based on the diagnosis of chorioamnionitis

Chorioamnionitis was detected in 413 of 7,727 pregnant women with premature rupture of membranes. The results are summarized in Table 2. There was no significant difference between the two groups in the maternal age at delivery, gestational week, educational level, previous history of preterm labor, pre-pregnancy BMI, and gestational diabetes mellitus (*p* > 0.05). In the chorioamnionitis group, the rates of complicated preterm delivery, maternal fever, postpartum hemorrhage, gestational hypertension, placental abruption, puerperal infection, neonatal hyperbilirubinemia, severe asphyxia of the newborn and admission to the NICU were higher than those of the control group (*p* < 0.05). According to the stratified analysis by gestational week of delivery, the incidence of preterm delivery at 34–36 weeks was significantly higher in the chorioamnionitis group than that in the control group (22% vs. 8%), WBC, NE and CRP counts were significantly higher than that in the control group, and the positive rate of cervical secretion cultures was significantly higher (34% vs. 23%). *Mycoplasma* and bacterial positivity were detected in 13% of all positive cultures, which was higher than that in the control group (8% and 6%, respectively), and the difference was statistically significant (*p* < 0.05).

**TABLE 2:** Baseline data and Maternal-Child Pregnancy Outcome based on the diagnosis of Chorioamnionitis.

Characteristics	No chorioamnionitis (n = 7,314)	Chorioamnionitis (n = 413)	*p*-Value
Age at delivery (y)	28 (26, 32)	28 (27, 32)	0.201
Education level [n (%)]			0.219
Primary school or below [n (%)]	50 (1)	4 (1)	
Junior and Senior high school [n (%)]	1,545 (25)	77 (21)	
College or higher [n (%)]	4,553 (74)	279 (78)	
Gravity			0.009
G < 3	5,388 (74)	322 (78)	
3 ≤ G < 5	1,665 (23)	70 (17)	
G ≥ 5	261 (4)	21 (5)	
Parity [n (%)]			<0.001
0	4,651 (60)	4,349 (59)	
1	2,888 (37)	2,785 (38)	
2	180 (2)	172 (2)	
3	7 (0)	7 (0)	
4	1 (0)	1 (0)	
Previous history of premature birth [n (%)]			0.169
0	7,137 (98)	398 (96)	
1	177 (2)	15 (4)	
Pre-pregnancy BMI (kg/m^2^)	20.31 (18.78, 22.19)	20.51 (19.14, 22.6)	0.049
Apgar score	10 (10, 10)	10 (10, 10)	<0.001
Gestational weeks	39.29 (38.29, 40.14)	39.29 (36.43, 40.29)	0.053
Premature birth (<37 w) [n (%)]			<0.001
0	6,668 (91)	292 (71)	
1	646 (9)	121 (29)	
Premature birth (<34 w) [n (%)]			<0.001
0	7,226 (99)	381 (92)	
1	88 (1)	32 (8)	
Delivery method [n (%)]			<0.001
CS	1,637 (22)	164 (40)	
VB	5,677 (78)	249 (60)	
Amount of postpartum hemorrhage (mL)	175 (145, 295)	237.5 (160, 415)	<0.001
Postpartum hemorrhage			0.039
0	7,180 (98)	399 (97)	
1	134 (2)	14 (3)	
Precipitate labor			0.095
0	6,590 (90)	383 (93)	
1	724 (10)	30 (7)	
GDM [n (%)]			0.81
0	6,029 (82)	338 (82)	
1	1,285 (18)	75 (18)	
HDP [n (%)]			0.043
0	7,084 (97)	392 (95)	
1	230 (3)	21 (5)	
Oligohydramnios [n (%)]			0.115
0	7,283 (100)	409 (99)	
1	31 (0)	4 (1)	
Polyhydramnios [n (%)]			0.648
0	7,290 (100)	411 (100)	
1	24 (0)	2 (0)	
Placenta previa [n (%)]			0.006
0	7,288 (100)	407 (99)	
1	26 (0)	6 (1)	
Placental abruption [n (%)]			<0.001
0	7,226 (99)	389 (94)	
1	88 (1)	24 (6)	
Puerperal infection [n (%)]			0.003
0	7,311 (100)	410 (99)	
1	3 (0)	3 (1)	
Neonatal jaundice [n (%)]			0.009
0	4,920 (67)	304 (74)	
1	2,394 (33)	109 (26)	
SGA [n (%)]			0.197
0	7,103 (97)	396 (96)	
1	211 (3)	17 (4)	
Mild asphyxia [n (%)]			<0.001
0	7,293 (100)	406 (98)	
1	21 (0)	7 (2)	
Serve asphyxia [n (%)]			0.05
0	7,309 (100)	411 (100)	
1	5 (0)	2 (0)	
NICU [n (%)]			<0.001
0	6,845 (94)	311 (75)	
1	469 (6)	102 (25)	
WBC	9.36 (7.86, 11.24)	10.68 (8.8, 13.27)	<0.001
NE	6.83 (5.56, 8.49)	8.23 (6.58, 10.96)	<0.001
CRP	2.2 (0.81, 5.03)	4.9 (1.22, 24.75)	<0.001
Culture of cervical secretion, [n (%)]			<0.001
negative	5,642 (77)	273 (66)	
mycoplasma	580 (8)	55 (13)	
germ	420 (6)	55 (13)	
fungus	610 (8)	27 (7)	
*chlamydia*	62 (1)	3 (1)	
Disease Type [n (%)]			<0.001
extremely preterm	3 (0)	9 (2)	
very preterm	37 (1)	17 (4)	
moderate preterm	48 (1)	6 (1)	
late preterm	558 (8)	89 (22)	
term labor	6,668 (91)	292 (71)	
Cervical secretion culture [n (%)]			<0.001
0	5,642 (77)	273 (66)	
1	1,672 (23)	140 (34)	
Membrane rupture to delivery time(h)	32.91 (19.7, 45.57)	33.79 (21.35, 46.08)	0.153
Fever (maternal temperature), [n (%)]			<0.001
0	7,271 (99)	388 (94)	
1	43 (1)	25 (6)	

G, gravity; BMI, body mass index; CS, cesarean section; VB, vaginal delivery; GDM, gestational diabetes mellitus; HDP, hypertension disorders of pregnancy; SGA, small for gestational age; WBC, white blood cell count; NE, neutrophil count; CRP, C-reactive protein.

Since cervical secretion cultures results differed between the two groups, logistic regression models were then constructed to adjust for confounders and to assess their value in predicting chorioamnionitis. After screening confounders based on the results of univariate analysis and clinical significance, cervical secretion cultures results, age, maternal fever, NE, Fever (maternal temperature), pre-pregnancy BMI, GDM, CRP, and WBC were included in the models (Table 3). Positive cervical secretion cultures results, WBC, and CRP were significantly associated with the risk of chorionic amnionitis in all three models (*p* < 0.05). The inclusion of cervical secretion culture as unordered multicategorical data in the model yielded results consistent with the univariate analysis. Specifically, a positive culture for *mycoplasma* or bacterial pathogens in cervical secretions remained an independent risk factor for chorioamnionitis, while positive cultures for fungi or *chlamydia* were not statistically significant.

**TABLE 3:** Odds ratio for chorioamnionitis, adjusted for cofounders.

	Model A		Model B		Model C	
	AOR (95%)	*p*-Value	AOR (95%)	*p*-Value	AOR (95%)	*p*-Value
Cervical Secretion Culture results	ref (negative)		ref (negative)		ref (negative)	
bacterial	2.687 [1.960, 3.625]	<0.001	2.594 [1.882, 3.516]	<0.001	2.274 [1.633, 3.113]	<0.001
chlamydia	1.002 [0.244, 2.724]	0.998	0.951 [0.228, 2.633]	0.934	0.967 [0.231, 2.691]	0.955
mycoplasma	1.965 [1.439, 2.638]	<0.001	1.887 [1.376, 2.544]	<0.001	1.765 [1.282, 2.390]	<0.001
fungla	0.916 [0.598, 1.346]	0.669	0.877 [0.569, 1.295]	0.528	0.820 [0.530, 1.217]	0.347
Age at delivery	1.008 [0.984, 1.032]	0.531	1.011 [0.987, 1.036]	0.365	1.014 [0.989, 1.039]	0.281
Pre-pregnancy BMI	1.029 [0.992, 1.066]	0.124	1.027 [0.989, 1.065]	0.159	1.016 [0.979, 1.054]	0.398
GDM			0.982 [0.747, 1.277]	0.897	0.988 [0.748, 1.290]	0.932
Membrane rupture to delivery time			1.004 [0.998, 1.011]	0.198	1.003 [0.997, 1.010]	0.342
Fever (maternal temperature)			10.290 [6.082, 17.059]	<0.001	6.867 [3.941, 11.690]	<0.001
NE					1.127 [1.089, 1.165]	<0.001
CRP					1.005 [1.002, 1.007]	<0.001
WBC					1.033 [1.000, 1.066]	0.042

GDM, gestational diabetes mellitus; CRP, C-reactive protein; WBC, white blood cell count; NE, neutrophil count.

Model A: adjustment was made for cervical secretion culture results, age, pre-pregnancy BMI, and Cervical Secretion Culture results.

Model B: AOR, increases GDM, Membrane rupture to delivery time and Fever (maternal temperature) for adjustment.

Model C: AOR, increases CRP, WBC, and NE, for adjustment.

### Value of CRP, WBC, maternal fever and cervical secretion cultures for predicting chorioamnionitis

The efficacy of CRP, WBC, maternal fever and cervical secretion cultures results in predicting chorioamnionitis was examined using ROC curves with the gold standard of a confirmed diagnosis of chorioamnionitis by placental chorioamnionitis pathology.

**FIGURE 2:**
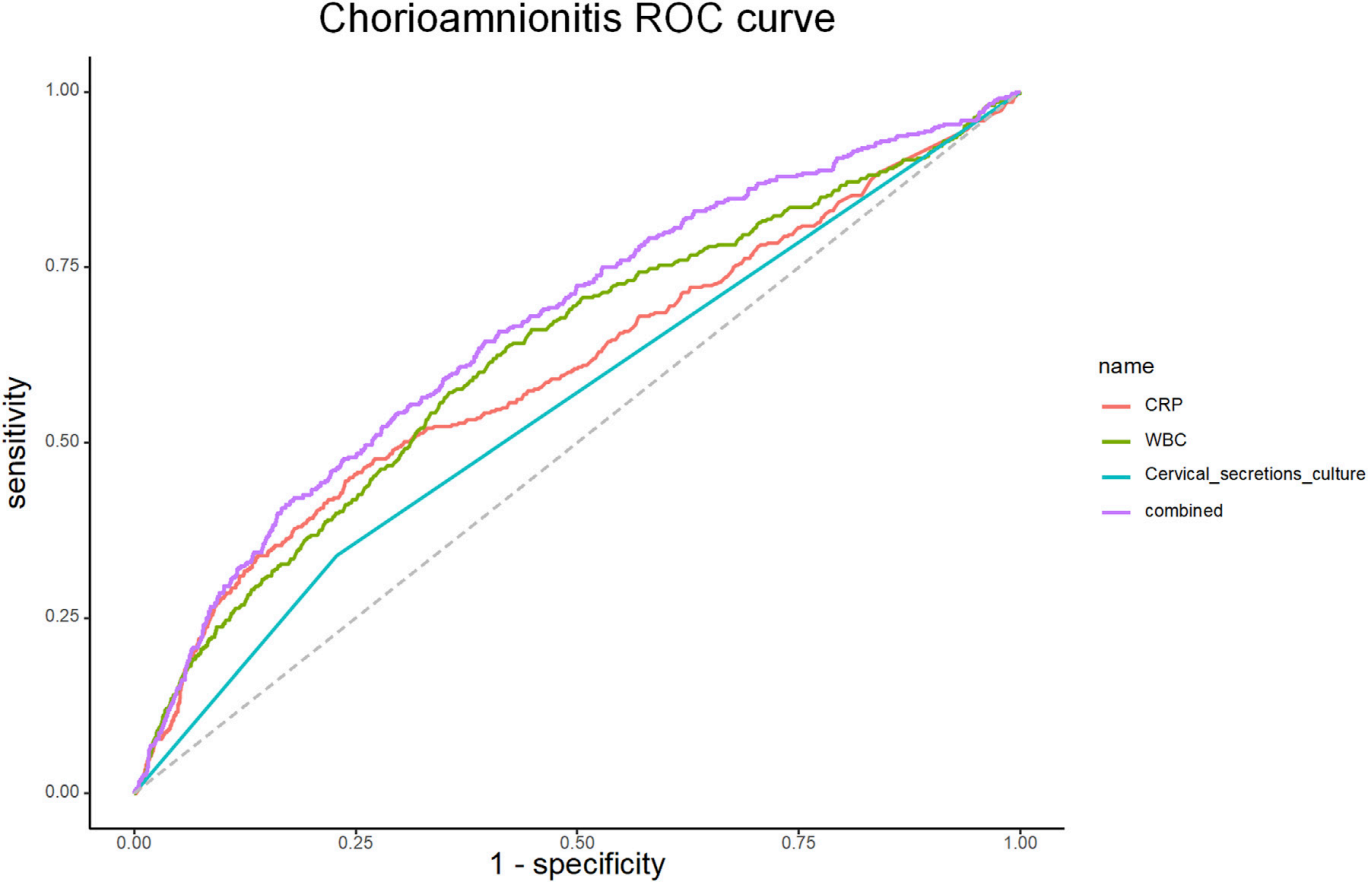
ROC curve for CRP, WBC, Cervical secretions culture and combination models.

The ROC curves were fitted according to the joint predictors, and the results showed that the AUC of the area under the curve of CRP, WBC, maternal fever and cervical secretion cultures results were 0.605 (95% CI 0.589-0.621), 0.626 (95% CI 0.611-0.641), 0.527 (95% CI 0.521-0.533) and 0.569 (95% CI 0.556-0.581), respectively. The joint predictors’ AUC was 0.687 (95% CI 0.673-0.701), and the predictive model IDI (Integrated Discrimination Improvement) was 0.0029, *p*-value: 0.338. (Table 4; Figure 2).

**TABLE 4:** Concordance index for predicting chorioamnionitis.

Characteristics	Concordance index (area under curve)	95% CI
CRP	0.605	0.589-0.621
WBC	0.626	0.611-0.641
Fever (maternal temperature)	0.527	0.521-0.533
Cervical secretion culture	0.569	0.556-0.581
CRP + WBC + Fever	0.673	0.659-0.688
CRP + WBC + Fever + Cervical secretion culture results	0.687	0.673-0.701

CRP, C-reactive protein; WBC, white blood cell count; NE, neutrophil count.

## Discussion

There is a clear connection between reproductive tract infections and premature rupture of membranes. Pregnancy is prone to changes in the vaginal microecology, which in turn causes changes in the long-term resident flora, leading to upstream infections and PROM. Prolonged PROM in turn increases the chances of chorioamnionitis complication which are associated with poor neonatal prognosis (Verma et al., 2023) and long-lasting effects such as impaired neuropsychological development of offspring (Gervasi et al., 2023). Several studies have shown that positive cervical secretion cultures in pregnant women with premature rupture of membranes are associated with the development of chorioamnionitis (Saghafi et al., 2018; Thakur et al., 2021). We found that cervical secretion cultures, although easy to perform, has a low predictive efficacy and is only useful as the diagnostic modality.

We showed a positive cervical secretion cultures detection rate of 23.45% in our PROM cohort, with an incidence of chorioamnionitis of 5.3%, which is broadly in line with the incidence of 1%–13% previously reported in the literature (Gibbs and Duff, 1991; Tita and Andrews, 2010; Jefferies, 2017; Spénard et al., 2023). Risk assessment analysis in our study demonstrated that bacterial and *Mycoplasma* infections were significant risk factors for chorioamnionitis. This association remained consistent even after adjusting for confounding variables.

The incidence of chorioamnionitis is increased in the presence of positive cervical secretion cultures and is associated with the development of puerperal infections and adverse pregnancy outcomes such as neonatal asphyxia and NICU admission. Chorioamnionitis complicated by fetal systemic inflammatory response syndrome is manifested by increased incidence of fetal intrauterine distress and neonatal asphyxia, as well as neonatal neurodevelopmental and chronic respiratory disorders. Some studies suggest that active prevention and treatment of chorioamnionitis may reduce the risk of intrauterine fetal infection and maternal puerperal infection (Sgayer et al., 2023).

WBC and CRP are commonly used in clinical practice as indicators of inflammation in response to local or systemic reactions. These indexes have high sensitivity and are mostly used to assess non-specific inflammatory states. Previous studies reported the use of WBC and CRP to predict unfilled chorioamnionitis (Galletta et al., 2023; Kong et al., 2023). In the present study, WBC and CRP were used as reference standards for the prediction of chorioamnionitis. Our results indicated that cervical secretion cultures had lower predictive value than WBC and CRP. This may be related to the presence of colonizing bacteria at the cervical opening, and suggests that the mechanism of chorioamnionitis is more complex than expected. Brandie DePaoli Taylor et al. found that gonococcal infection was directly associated with poor neonatal prognosis, and that subclinical intrauterine infections and localized inflammatory reactions in the fetal membranes near the cervical os may be the main cause of chorioamnionitis (Taylor et al., 2023). In another study, Stepanovich, Gretchen E et al. found that the impact of chorioamnionitis on the prognosis of newborns is linked to the local immune response (Stepanovich et al., 2023). In addition, it is important to keep in mind the ability of the placenta to clear inflammatory response factors and to maintain local immune balance homeostasis (Mir et al., 2023). Moreover, the analysis of the cervical specimen may be influenced by the amount of amniotic fluid flow. Therefore, a positive result does not necessary indicate the upstream infection with resident vaginal microorganisms. Alternatively, it may indicate an intrauterine infection in which the bacteria reside in the cervix as the amniotic fluid flows out. Similarly, culture results may be negative because of the early stage of bacterial infection or because of bacterial species that are difficult to culture (Berezowsky et al., 2022).

Li, et al. (Li et al., 2022) sequenced vaginal and cervical secretions from women with normal full-term pregnancy, clinical chorioamnionitis and histological chorioamnionitis, respectively, and showed that these groups had similar cervical microbiological results. Germano, et al., on the other hand, found that vaginal colonisation with *Mycoplasma* and *Mycoplasma* Ureaplasma during pregnancy was associated with poor neonatal outcome, whereas there was no significant difference in neonatal outcomes in women with *Candida* infection (Germano et al., 2022). This also highlights the limited value of cervical secretion culture results alone in predicting chorioamnionitis. A wide range of bacteria are naturally colonizing both infected and non-infected cervical surfaces. There may be unique microbiological profiles of bacteria in the membranes following the initiation of labor or PROM, and additional methods of greater sensitivity and specificity may be required to identify the pathogens responsible for amniotic cavity infections to assist in the development of a targeted therapeutic strategy (Sayres et al., 2023).

Bacterial culture of amniotic fluid, taken by amniocentesis in patients with clinically diagnosed chorioamnionitis, and vaginal secretion testing revealed homologous bacterial profiles that predominantly contained *Mycoplasma*, *Escherichia coli*, and *Lactobacillus* (Romero et al., 2019), which is consistent with the predominance of *Mycoplasma* and bacteria in the culture of cervical secretions in the present study. Vaginal upstream infection is an important route of infection in chorioamnionitis. However, vaginal microecology tests were not done in our study. Therefore, there is a possibility that vaginal bacteria may have influenced the culture results. In addition to genital tract epithelial infections, mother-to-child vertical infections such as rubella virus and cytomegalovirus may be transferred to the fetus via the placenta. Additionally, recent studies reported a presence of pathogenic bacteria associated with periodontal disease in amniotic fluid (Miranda-Rius et al., 2023), suggesting that hematogenous dissemination may also be an etiological factor leading to chorioamnionitis. This partly explains the limitations of the method of bacterial culture using only cervical secretions, which does not cover all causes of localized placenta-placenta inflammation.

PROM is a syndrome of multiple etiologies and pathways. The leading causes of rupture of membranes, other than infection, may vary from gestational week to gestational week. In mid-gestation or during very early preterm labor, the cause of rupture of membranes may be related to cervical insufficiency, whereas in mid to late preterm labor it may be related to untreated chronic infections. In full-term pregnancies rupture of membranes may be related to an increase in pressure in the amniotic cavity, e.g., in twin pregnancies, excess amniotic fluid, placenta previa, and hypertension in pregnancy. Therefore, clinical decision-making for PROM should not just consider gestational week and infection, but also take into account etiology and comorbidities. This study was a study of singleton PROM pregnancies, not stratified according to complications. Pregnant women with clinically typical chorioamnionitis and multiple births were excluded. Therefore, based on the results of cervical secretion cultures we were able to predict tissue chorioamnionitis at the level of infection of the reproductive tract, while it was not possible to predict placenta-derived complications due to non-infectious factors, such as failure of physiological transformation of the spiral arteries of the myometrium, as well as vertical transmission leading to placenta infections and inflammatory reactions. A mean gestational age of study participants was 39 weeks, with only few women who were not full term. Women were not stratified according to the gestational week of rupture, and only the results of a single culture of cervical secretion was included in the analysis for women who were not full term. This may also explain the poor performance and has limited value of cervical secretion culture in predicting the occurrence of chorioamnionitis in women with PROM and the under-term pregnancy.

Considering the high cost of a single cervical secretion culture, its limited performance in predicting chorioamnionitis, the average time to culture results (2–5 days), a need for regular monitoring of peripheral blood inflammation indicators, and a little significance of the test in clinical decision-making for the management of PROM, we suggest that the test may only be used as an optional method in economically developed areas.

There are some limitations for this study. This is a single-center retrospective cohort study. Only the vaginal route of infection was analyzed, the sampling time of cervical secretion specimens was not standardized, and the vaginal secretions were not sampled for culture at the same time. Additionbally, no stratification of gestational weeks, complications, and PROM in the first trimester of pregnancy was done. The neonatal outcomes lacked long-term observational indicators such as the postnatal period of 14 days and neurodevelopmental indicators for follow up. A multi-centre, prospective cohort study is needed to add other simple inflammatory indicators for clinical prediction of chorioamnionitis in order to guide clinical therapeutic decisions.

## Conclusion

Positive cervical secretion cultures, especially for *mycoplasma* and bacteria, are associated with higher rate of adverse maternal and fetal outcomes but are not good predictors of chorioamnionitis. Further predictive models that incorporate peripheral blood and other indicators are needed.

## Data Availability

The original contributions presented in the study are included in the article/Supplementary material, further inquiries can be directed to the corresponding author.
